# Field evaluation of an automated RDT reader and data management device for *Plasmodium falciparum/Plasmodium vivax* malaria in endemic areas of Colombia

**DOI:** 10.1186/1475-2875-13-87

**Published:** 2014-03-10

**Authors:** Sócrates Herrera, Andrés F Vallejo, Juan P Quintero, Myriam Arévalo-Herrera, Marcela Cancino, Santiago Ferro

**Affiliations:** 1 Caucaseco Scientific Research Center, Cali, Colombia; 2 Latin American Center for Malaria Research, Cali, Colombia; 3 Faculty of Health, Universidad del Valle, Cali, Colombia; 4 Fio Corporation, Toronto, Canada

**Keywords:** DekiReader, Malaria rapid diagnostic test, PCR, mHealth

## Abstract

**Background:**

Massive implementation of malaria diagnostics in low-resource countries is regarded as a pivotal strategy in control and elimination efforts. Although malaria rapid diagnostic tests (RDTs) are considered a viable alternative, there are still obstacles to the widespread implementation of this strategy, such as reporting constraints and lack of proper quality assurance of RDT-based programmes at point-of-care (POC).

**Methods:**

A prospective cohort of patients, seeking routine care for febrile episodes at health centres in malaria-endemic areas of Colombia, was used to assess the diagnostic performance of a device based on smartphone technology (Deki Reader^TM^, former codename “GenZero”), that assists users at POC to process RDTs. After informed consent, patients were enrolled into the study and blood samples were collected for thick blood smear (TBS) and RDT. The RDT results were interpreted by both visual inspection and Deki Reader device and concordance between visual and device interpretation was measured. Microscopy corrected by real-time polymerase chain reaction (PCR) and microscopy were “gold standard” tests to assess the diagnostic performance.

**Results:**

In total, 1,807 patients were enrolled at seven health centres in malaria-endemic areas of Colombia. Thirty-three *Plasmodium falciparum* and 100 *Plasmodium vivax* cases were positive by corrected microscopy. Both sensitivity and specificity were 93.9% (95% CI 69.7-95.2) and 98.7% (95% CI 98.5-99.4) for *P. falciparum,* and 98.0% (95% CI 90.3-98.9) and 97.9% (95% CI 97.1-98.5) for *P. vivax*. Percentage concordance between visual and device interpretation of RDT was 98.5% and 99.0% for *P. vivax* and *P. falciparum*, respectively.The RDT, when compared to TBS, showed high sensitivity and specificity for *P. falciparum* in both visual and device interpretation, and good overall diagnostic performance for *P. vivax*. Comparison between PCR as gold standard and visual and device interpretation showed acceptable overall performance for both species.

**Conclusions:**

The diagnostic performance of the Deki Reader was comparable to visual interpretation of RDTs (without significant differences) for both malaria species. Providing standardized automated interpretation of RDTs at POC in remote areas, in addition to almost real-time reporting of cases and enabling quality control would greatly benefit large-scale implementation of RDT-based malaria diagnostic programmes.

## Background

Malaria is transmitted in about 106 countries in tropical and subtropical regions where ~3.3 billion people, half of the world’s population, are at risk of malaria transmission. The World Health Organization (WHO) estimates that over 219 million cases (uncertainty range of 154 to 289 million) and about 660,000 malaria deaths (uncertainty range of 490,000 to 836,000) occur worldwide every year [1], representing an enormous global social and economic burden. Clinical manifestations of the disease are similar to many other infectious diseases making clinical diagnosis alone imprecise and inaccurate. Confirmation of malaria, which depends on the detection of *Plasmodium* parasites in blood circulation, is not only critical for appropriate case management [2], but it is essential for epidemiological purposes and to enhance malaria control programmes.

Microscopic detection of *Plasmodium* species on thick blood smear (TBS) has been for decades the standard method for malaria diagnosis despite its field accuracy, errors in species identification and its operator-dependence. Although it is less expensive and easier to handle than other malaria diagnostic methods developed more recently, it is laborious and time consuming, and it requires electricity as well trained personnel, frequently not available in remote malaria-endemic areas. Recently, a number of techniques aimed at reducing the time of processing and some other constraints of TBS have been developed. Some of them include fluorescence stains but still require microscopes, others are based on detection of parasite nucleic acids in human blood circulation but require expensive and complex equipment, and others are based on the use of antibodies that recognize parasite components [3,4]. The methods based on parasite nucleic acid detection, have proven great sensitivity and specificity, however, they require significant infrastructure and training, and are significantly more expensive than TBS, therefore precluding expanded implementation in low-resource settings [5]. Attempts to simplify these methods are currently underway based on loop-mediated isothermal amplification (LAMP) which requires less sophisticated equipment, such as a water bath and ultraviolet light, which could be installed in more underserved regions, but still requires electricity [6].

Malaria diagnostic methods based on the use of monoclonal antibodies recognizing parasite antigens [3,4] have been grouped under the name of malaria rapid diagnostic tests (RDT). These methods have shown great performance in term of sensitivity and specificity, although they still present limitations, such as batch-to-batch variation, and limited sensitivity at low parasitaemia levels (<0.1%). Although these RDTs still require improvement, their apparent simplicity currently makes them attractive to provide diagnosis at all levels of the health care system, particularly in remote areas where health workers have limited supervision and training.

The reliability of RDT results is pivotal to ensure the safety of withholding anti-malarial treatment in test-negative patients [7-10]. As recommended by WHO, the possibility of diagnosis error using RDTs can be minimized through a careful selection process [11]. Important steps involved in the process of ensuring high quality of RDTs include among others, high diagnostic performance as assessed in product testing by the WHO and the Foundation for Innovative New Diagnostics (FIND) [12], proper storage of the test kits, adequate labelling, clear operation instructions, continuous on-the-job training (job aids), and automated interpretation to avoid inter-user variability. In addition, implementation of large-scale diagnostic programmes based on malaria RDTs requires the establishment of a comprehensive quality assurance (QA) strategy. This task can be particularly challenging considering that most RDTs are being used at point-of-care (POC) in health centres and dispensaries located in remote areas.

The performance of health professionals using RDTs can be improved through simple redesign of job aids [7,8,13,14], as well as by QA programmes based on supervisory visits to assess their competence to correctly use and interpret RDTs results [15]. However, the sustainability of this type of QA programme is highly questionable due to costs of personnel, transportation and other expenses [16,17]. Currently available technologies such as cell-phone networks, could reduce costs and increase the efficiency as a supervisor could conduct QA activities to multiple facilities without the need to visit the sites, as shown in preliminary studies with both malaria and HIV RDT programmes [18-20].

The present report describes a field trial in malaria-endemic areas of Colombia of a system that includes mobile, battery-operated devices based on smartphone technology that assists users at POC to process RDTs, performing automated interpretation, reducing notification time to a cloud-based database and allowing malaria case geo-location and remote QA activities. The main objective of the trial was to assess the concordance between automated interpretation of RDTs by the device and by visual inspection by laboratory experts.

## Methods

### Study design and study sites

**Figure 1:**
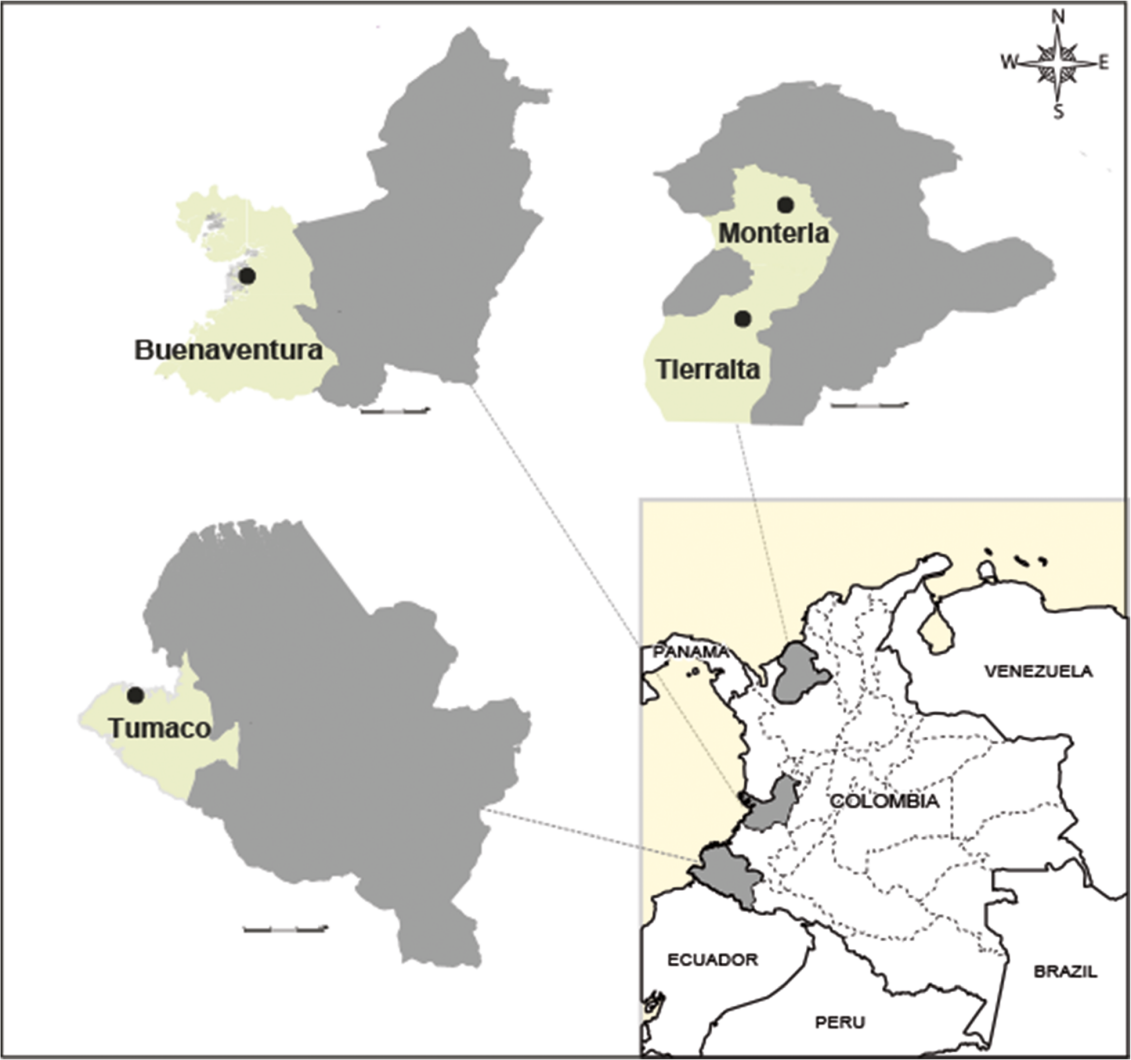
Geographical location of sites participating in the field study.

The present study involved a prospective cohort of consecutive patients from August 2011 to January 2012 at seven sites in four towns located in malaria-endemic areas of Colombia (Figure 1): Tumaco and Buenaventura in the Pacific coast, and Monteria and Tierralta in the north-western department of Córdoba. These four sites were selected based on their high malaria incidence and their reliable mobile phone connectivity. Patients were recruited at the main governmental malaria diagnosis facility, regional hospitals and Public Health Laboratory and at a diagnostic centre established by Caucaseco Scientific Research Centre (Caucaseco). Tumaco, is a municipality situated close to the border with Ecuador, in the southwest area of Colombia with a population of 100,000 inhabitants and with *Plasmodium falciparum* as predominant parasite species. Buenaventura is the main port of Colombia on the Pacific coast with a population of ~360,000 habitants, with similar prevalence of both *P. falciparum* and *Plasmodium vivax* infections. Monteria is the political capital of the department of Córdoba, with a population of ~300,000 inhabitants and Tierralta, a town located about 100 km south of Monteria, with a population of ~80,000, with predominance of *P. vivax* infections. Patients were mostly Afro-descendants of both genders, seeking routine care for febrile episodes and were enrolled into the study after signing an informed consent form. Before the conduct of the trial, the protocol was reviewed and approved by the institutional ethics committee (CECIV, May 2011, Code 008).

### Study population

Eligible participants were consecutively enrolled in the dispensaries and at the outpatient clinics of the institutions described above. Inclusion criteria were age ≥ one year, a documented fever or a history of fever in the previous 48 hours and a written informed assent/consent to participate in the study. No information regarding recent use of anti-malarials was collected. Study technicians enrolled study participants and collected demographic and clinical information electronically in the device using a structured form. After enrolment, blood was collected once from a finger prick (no follow-ups) and about three to four blood drops were used for preparation of TBS, RDTs, and a filter-paper blood spot for real-time polymerase chain reaction (PCR). The latter was performed as quality control of microscopy. Technicians working at the reference laboratory were blinded to results of both visual and device interpretation of RDTs performed at the site. Likewise, results of TBS microscopy examination and PCR (when applicable) were blinded to site technician who performed the RDTs. The processing of the RDT was performed according to manufacturer recommendations and guided by a job aid displayed on the screen of the Deki Reader device (former codename “GenZero”). The device also assisted technicians at the sites in keeping track of the incubation time for each RDT processed. Once the incubation period was completed, the study technician interpreted the RDT by visual inspection of the strip and captured the result using the touch-screen of the device. The study technician then immediately inserted the cassette into the device to perform the automated interpretation of the test. The results of the device’s interpretation of the RDT were concealed at all times, and were generated in all cases after the study site technician had entered results of her/his visual interpretation of RDT test results. All results of RDTs interpretation, along with a high-resolution image of the RDT captured by the device, and the patient data collected were encrypted and automatically transmitted to a central cloud database specifically designed for the purpose of the trial. Patient management was performed according to official protocols of the national malaria control programme [21], based on the results of TBS microscopic examination.

### Laboratory analyses and device procedures

#### Microscopic diagnosis

Symptomatic patients were bled by finger prick and TBS were prepared and stained using the standard 10% Giemsa method [22]. Slides were examined under high power objective and asexual parasites were counted against 200 white blood cells (WBCs). Smears were considered negative if the examination of 200 high power fields did not reveal asexual parasites.

#### Malaria RDTs

The malaria RDT used in the current study was SD Bioline Malaria Antigen Pf/Pv (Catalogue No. 05FK80, Standard Diagnostics Inc., Hagal-Dong, Korea), referred here to as RDT. This is a lateral flow immunochromatographic test that contains a membrane strip encased in a flat plastic cassette. The strip is pre-coated with two monoclonal antibodies: one specific for *P. falciparum* HRP2 protein and the other one specific for *P. vivax* pLDH enzyme. Interpretation of the results was based first on the presence of the control line, and then on the presence of one or two lines.

#### PCR correction

PCR was adapted from Rougemont *et al.*[23], and performed in all cases of discrepant results between expert visual interpretation of RDT, device’s interpretation of RDT and expert microscopy results (tie-breaker). Genomic DNA was extracted from dried filter paper spots using Chelex-resin (Biorad, Hercules, CA, USA) according to the method of Wooden *et al*. [24]. PCR was performed using the same set of primers and species-specific probes (Applied Biosystems, USA), with reporters and quenchers adapted to the dye channels of ABI7500 device (Applied Biosystems, USA). Duplex PCR reactions were run in parallel to detect *P. falciparum* and *P. vivax*, the 20 μl reaction mix contained 2 μl DNA, 1× TaqMan Master Mix (Applied Biosystems, USA), 200 nM forward and reverse primer, 200 nM *P. falciparum* probe and 100 nM *P. vivax* probe. The PCR programme consisted of an initial step of 10 min at 95°C followed by 40 cycles of 10 sec at 95°C and finally 60 sec at 60°C. Each experiment included the test sample in duplicate, non-infected DNA as negative control and a positive control with a known parasitaemia for *P. vivax* and *P. falciparum*. Parasitaemia was calculated using an average standard curve of pre-determined parasitaemia. The detection limit calculated was one parasite/ml based on the upper limit of the calibration curve at the lowest detected concentration. In addition, PCR was performed in a randomly selected subset of samples, and used in an *ad hoc* analysis with PCR results as gold standard (n = 274).

### The Deki Reader™ (formerly known with codename “GenZero”)

**Figure 2:**
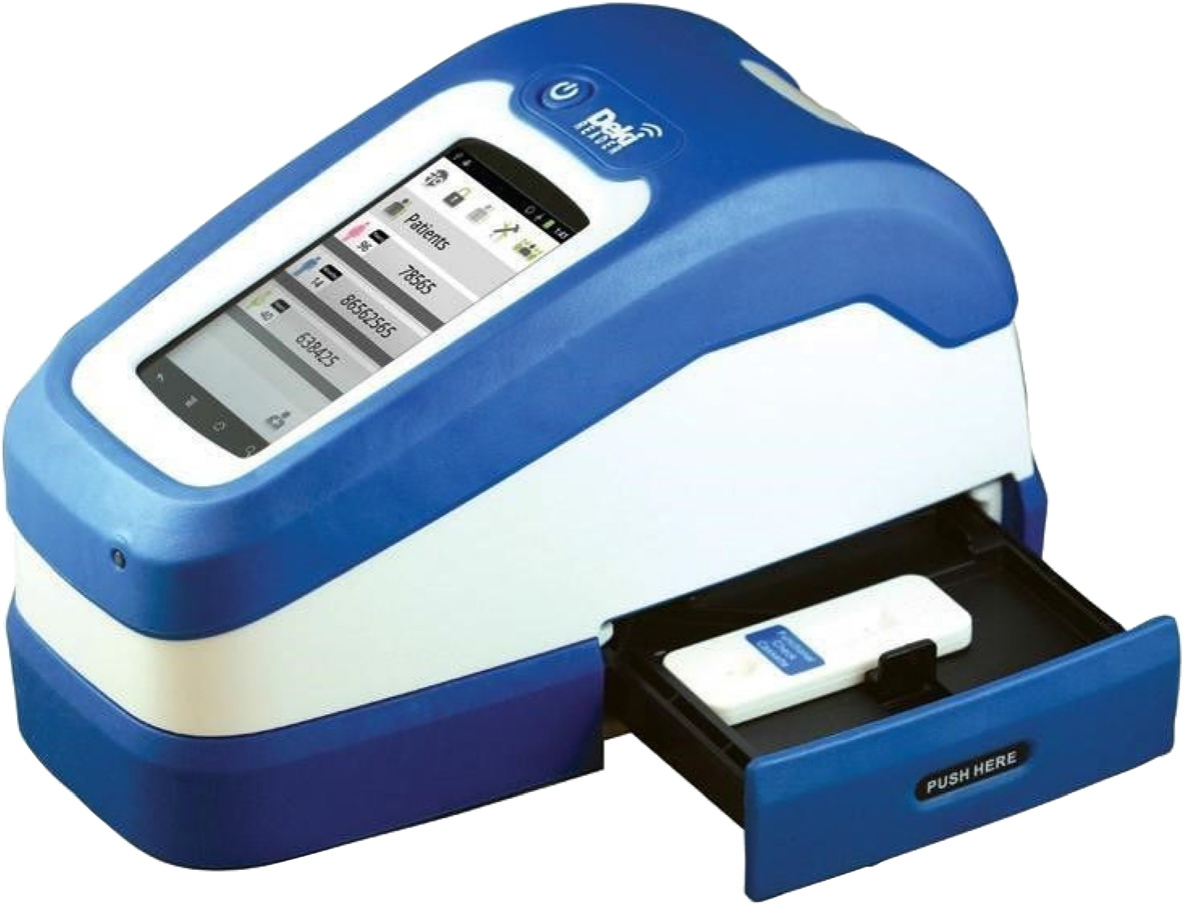
Deki Reader device.

This is a mobile, ruggedized, battery-operated device (Figure 2), manufactured by Fio Corporation, (Toronto, Canada), designed to perform the following functions: 1) automated interpretation of RDTs by means of image analysis software; 2) digital data capture, by means of a touch-screen and a simple user interface software; and, 3) transmission in real-time (RT) of all data to an accessible central database, using local mobile phone network. Transmitted data included: processed RDT image, diagnostic event data collected, user and device interpretation of test results, geo-positioning of the device, and date and time stamp. The principal investigator (PI) and study coordinators had permanent access to the database via the internet portal especially designed for the study.

### Training on the operation of Deki Reader

Seven laboratory microbiologists with five years of university education and former training on malaria microscopy were engaged to operate the Deki Reader and to fill up all required documentation for the study. These laboratory personnel were trained in a two-day workshop on all study procedures, the operation of RDTs assembled to a Deki Reader, as well as on the collection of patients’ information, including the informed consent form.

### Statistical methods

The prevalence of malaria cases (*P. falciparum* and *P. vivax*) at the study areas was estimated to be 10 to 15%, *P. vivax* being responsible for ≥75% of the malaria cases. Based on this prevalence, the sample size was estimated to be between 1,800 to 2,000 subjects, under the assumption that it would provide approximately 120 to 225 positive results for *P. vivax* and 40 to 75 positive results for *P. falciparum*. Assuming that the sensitivity and specificity of the interpretation of RDT is the same as the human-eye interpretation, this sample size would provide 95% confidence intervals within 5% of the point estimate. Results of RDT were interpreted by both visual examination and by the Deki Reader device.

Data were obtained from the source documents at each of the participant malaria sites and was compared with the information collected through devices and transmitted to the cloud database. Data collected were monitored according to U S Food and Drug Administration (FDA) regulations and Good Clinical Practice (GCP) guidelines.

There were three outcomes of interest for the purpose of the analysis in the current trial: a) microscopy results: performed on TBS by laboratory technician at reference laboratory and entered manually into the dataset; b) visual interpretation of RDT: performed by laboratory technician at the site following visual inspection of the RDT at the appropriate time, and entered into the Deki Reader device; and, c) device interpretation of RDT: performed by the device, which was automatically collected and transmitted to the database by the device. Data collected at the cloud database were reconciled to data collected in the internal memory card of each Deki Reader device to ensure that all data points were correctly transmitted to the cloud database. Data analysis was performed using JMP Version 8.0.2 and R Version 2.12.1 (SAS Institute Inc. NC, USA). The primary analysis was conducted using microscopy corrected by PCR as gold standard to determine diagnostic performance characteristics [25,26] (sensitivity, specificity, negative (NPV) and positive (PPV) predictive values, and overall diagnostic accuracy) of RDT interpretation by the Deki Reader and by visual interpretation. Secondly, microscopy and PCR were used as gold standards. Correspondent 95% confidence intervals were constructed using the Wilson score method. The percentage of agreement in the interpretation of RDTs between the device and visual (human-eye) done by the expert laboratory technicians at the sites was calculated as percentage of negative agreement, percentage of positive agreement and overall percentage of agreement. Two-sided 95% confidence intervals were constructed using the Wilson score method. Fisher’s exact test was used to compare differences between device and visual interpretation and P value < 0.05 was considered statistically significant.

## Results

**Figure 3:**
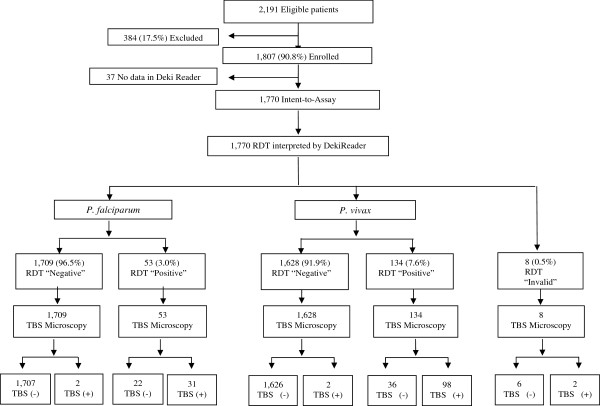
Patient flowchart for Deki Reader RDT results.

Between August 2011 and January 2012, a total of 2,191 subjects were screened. However, the final analysis included 1,807 samples, after 384 (17.5%) were excluded due to lack of compliance with the protocol: 238 (62%) were samples collected in a centre where RDT processing was performed in major violation of RDT manufacturer’s recommendation; 78 (20%) patient samples due to incomplete written informed consent process, and 68 (18%) due to RDT processing by non-study authorized personnel (Figure 3).

The main demographic features of the cohort are described in Table 1. The proportion of female participants was 54.3%, and the population was predominantly adults (76.4%) between 17 and 65 years of age. The prevalence of microscopically confirmed malaria cases among the recruited patients was 7.58% (33 cases of positive *P. falciparum* infection and 100 cases of *P. vivax*). Positive cases varied among the sites between 1.89 and 5.84% for *P. faciparum*, and between 1.52 and 10.54% for *P. vivax,* which is in agreement to the local known epidemiology concerning distribution of *Plasmodium* species infections [27]. Prevalence of *P. falciparum* infections was higher in the sites of Tumaco and Buenaventura (25 *P. falciparum* and 30 *P. vivax* infections), while *P. vivax* infection was higher in Tierralta (eight *P. falciparum* and 70 *P. vivax* infections).

**Table 1:** Demographics of study population

**Enrolled (N = 1,807)**	
Gender	
Female	982 (54.3%)
Male	825 (45.7%)
Age (years)	
Mean (SD)	30.74 (17.05)
Median	27.92
Range	[0.2, 88]
Age categories (years)	
<1	16 (0.9%)
≥ 1 to < 17	341 (18.9%)
≥ 17 to <65	1,380 (76.4%)
≥ 65	70 (3.8%)

Of the 2,191 samples, in 37 (1.7%) no primary or secondary outcomes (regarding results of the interpretation of RDTs), either visual or by the device were found in the dataset, most likely due to software malfunction, and these patients were not included in the analysis. Therefore, the final analysis dataset consisted of 1,770 samples (Figure 3).

### Comparison between diagnostic performance characteristics of the visual interpretation and Deki Reader interpretation of RDTs

The percentage agreement between visual interpretation and Deki Reader interpretation of RDTs for *P. falciparum* was 95.7% (95% CI 85.5-98.8) when a positive result was obtained, 99.1% (95% CI 98.5-99.4) when a negative result was obtained, and an overall percentage agreement of 99.0% (95% CI 98.4-99.4) (Table 2). The percentage interpretation agreement between the Deki Reader and visual for *P. vivax* was 98.9% (95% CI 94.0-99.5), when a negative result was obtained; 99.1% (95% CI 97.8-99.0) when a positive result was obtained; and an overall agreement of 98.5% (95% CI 97.9-99.0). There was no statistically significant difference in the sensitivity or in the specificity between the two methods of interpretation for any of the *Plasmodium* species.

**Table 2:** Comparison of performance between Deki Reader and visual interpretation (n = 1,762) of RDTs

**Parasite species**	**Negative % agreement (95% CI)**	**Positive % agreement (95% CI)**	**Overall % agreement (95% CI)**
*P. falciparum*	99.1 (98.5 - 99.4)	95.7 (85.5 - 98.8)	99.0 (98.4 - 99.4)
*P. vivax*	98.9 (94.0 – 99.5)	99.1 (97.8 - 99.0)	98.5 (97.9 - 99.0)

### Discordant results between Deki Reader and visual interpretation of RDTs

From a total of 30 discrepant test results, discordant results were obtained in 26 RDTs (1.47%) for either *P. falciparum* or *P. vivax* results and two showed discordant results for both *Plasmodium* species. In 28/30 (93%) discrepant interpretations, the visual inspection failed to detect a line, but the device reported a “positive” result. In the remaining two cases, it was the opposite. In ten (33%) of the 30 discrepant results, a closer review of the images of the RDTs revealed the presence of artefacts and bizarre smearing of the test strip that could have caused the software in the device to interpret as presence of a line. The presence of these unusual patterns in the flow of the sample along the diagnostic strip renders the test inconclusive and therefore should have resulted in processing a new RDT. In an additional seven cases, including the two where the visual inspection reported the presence of a line, the discrepancy seems to be originated from clerical mistakes by either registering a negative result when clearly visible lines were present in the RDT, registering a positive result when no lines were visible and no parasites were reported either by microscopy or PCR, or by mistakes in the identification of the tests. In an additional three cases, results of microscopy and/or PCR confirmed the presence of parasites and therefore were true positive cases (device interpretation was correct). In the remaining ten cases, all of which the device interpreted as positive with line intensity values very close to the cut-off value, both microscopy and PCR failed to detect presence of parasites and therefore correspond to false positive results (0.56%) (device interpretation was incorrect).

### Sensitivity and specificity of Deki Reader and visual interpretation of RDTs

Of the 1,770 samples tested, 94 were positive for *Plasmodium* by both diagnostic methods (TBS and RDT), 1,525 were negative by both diagnostic tests and 132 had discordant results. Of these discordant samples, 70 (4%) were positive only by RDT, 43 (2%) were positive only by TBS and 19 (1%) had discordant parasite species results. This discrepancies were solved using PCR as a tie-breaker, finding that 24 (34%) of the 70 RDT-positive samples were positive, 39 (90%) of the 43 TBS-positive samples were negative and 74% (14/19) the samples with species discrepancies had PCR results that were in line with RDT results.

### TBS microscopy as gold standard

When standard TBS microscopy results were compared to interpretation of RDT, the Deki Reader had a sensitivity of 33.3% (95% CI, 21.4-47.6) and a specificity of 98.1% (95% CI 97.3-98.7) for detection of *P. falciparum* infections, and sensitivity of 72.1% (95% CI, 62.5-80.5) and a specificity of 96.5% (95% CI 95.6-97.4) for detection of *P. vivax* infections. The correspondent sensitivity of expert visual interpretation of RDTs was 35.1% (95% CI, 22.9-48.9) and specificity of 98.4% (95% CI 97.7-98.9) for detection of *P. falciparum* infections, and sensitivity of 71.8% (95% CI, 62.1-80.3) and a specificity of 97.4% (95% CI 96.5-98.0) for detection of *P. vivax* infections (Table 3).

**Table 3:** Deki Reader diagnostic performance against microscopy

**Microscopy**		**Sensibility (%)**	**Specificity (%)**	**PPV (%)**	**NPV (%)**	P-Value*
*P.vivax*	Visual	71.8	97.4	62.7	98.3	0.876
	Device	72.1	96.6	56.0	98.3	
*P.falciparum*	Visual	35.1	98.4	41.7	97.9	0.843
	Device	33.3	98.1	35.9	97.8	

*Fisher’s exact test for sensibility.

### TBS microscopy corrected by PCR as gold standard

When compared to microscopy corrected by PCR, the Deki Reader had a sensitivity of 93.9% (95% CI 69.7-95.2) and a specificity of 98.7% (95% CI 98.5-99.4) for detection of *P. falciparum* infections, and sensitivity of 98.0% (95% CI 90.3-98.9) and a specificity of 97.9% (95% CI 97.1-98.5) for detection of *P. vivax* infections (Table 4). The sensitivity and specificity of expert visual interpretation of RDTs were identical to the Deki Reader for detection of *P. falciparum* infections, and for detection of *P. vivax* infections the sensitivity was 87.8% (95% CI 86.4-97.2) and the specificity 99.0% (100% CI 98.0-99.14). Finally, the performance of microscopy was 60.6% (95% CI 44.9-78.5) and 98.0% (95% CI 97.3-98.6) for sensitivity and specificity against *P. falciparum*; and 75.0% (95% CI 66.0-83.4) and 98.4% (95% CI, 97.7-99.0) for sensitivity and specificity against *P. vivax* (Table 4). The diagnostic performance of visual and Deki Reader interpretation of RDTs was virtually identical, and showed a higher sensitivity than microscopy for both species for all the gold standards used.

**Table 4:** Deki Reader diagnostic performance against microscopy corrected by PCR

**Microscopy corrected by PCR**		**Sensibility (%)**	**Specificity (%)**	**PPV (%)**	**NPV (%)**	P-Value*
*P. vivax*	Visual	94.0	98.6	80.5	99.6	0.149
	Device	98.0	97.8	73.1	99.8	
*P. falciparum*	Visual	87.8	99.0	62.5	99.7	0.392
	Device	93.9	98.7	62.5	99.7	

*Fisher’s exact test for sensibility.

### PCR as quality control for microscopy

When compared to PCR results performed in a subset of 274 randomly selected samples the performance of microscopy was 54.6% (95% CI 23.4-83.3) and 98.9% (95% CI 96.7-99.8) for sensitivity and specificity against *P. falciparum*; and 68.0% (95% CI 46.5-85.0) and 98.8% (95% CI 96.5-99.75) for sensitivity and specificity against *P. vivax* (Table 5).

**Table 5:** Quality control of microscopy using PCR on a subset of samples

**PCR**	**Sensibility (%)**	**Specificity (%)**	**PPV (%)**	**NPV (%)**
*P. vivax*	68.0	98.8	85.0	96.9
*P. falciparum*	60.6	98.0	66.7	98.1

## Discussion

Early and adequate malaria diagnosis is critical to opportune and effective treatment to prevent clinical complications and death. Among the malaria diagnostic tools currently available, microscopic examination of TBS remains the gold standard for defining malaria infection, not only because of the relative ease and low cost, but also for its ability to provide a quantitative result. Malaria RDTs are particularly useful in remote areas of difficult access and lack of basic infrastructure required in a microscopy post. The interpretation of two test lines contained in the RDT may be challenging from the point of view of personnel with low level of education, training and supervision (i e, community health workers) located in remote rural areas. Although the SD Bioline Malaria Antigen Pf/Pv RDT imposes this difficulty, it was decided to use this RDT in combination with the Deki Reader due to the known presence of both parasite species in the study areas. A previous study conducted in rural health facilities in Tanzania demonstrated that interpretation of SD Bioline Malaria Ag Pf/Pan RDT by Deki Reader is substantially equivalent to that of visual interpretation as performed by laboratory technicians with vast experience in RDT processing and interpretation [28], suggesting that a device such as the Deki Reader can be used in routine care for interpretation of RDT with high-quality diagnostic results at POC.

In the present study, the performance of the Deki Reader interpreting SD Bioline *Pf/Pv* RDTs was at least as good as the visual interpretation by experts, with sensitivity, specificity, positive and negative predictive value similar to visual interpretation of the same RDT (Tables 2 and 3). There was no statistically significant difference between the diagnostic accuracy of the Deki Reader and the visual interpretation of RDTs (Fisher’s exact test).

Important differences in the accuracy of the Deki Reader were identified when microscopy was used as the gold standard for malaria diagnosis. Most of the RDT-positive/microscopy-negative discordance is likely due to reduced specificity as result of the persistent antigenaemia of *P. falciparum* for approximately two weeks after parasite clearance [29] and most of microscopy-positive/RDT-negative cases might have originated in deficiencies in the microscopy test.

Similar findings to the present study with respect to comparative performance of microscopy *versus* RDTs have been reported previously [30-35], and the sensitivity and specificity of the RDTs with this reference test were in agreement with previous reports, including the third evaluation round of FIND [12]. In the same way, other studies using microscopy corrected by PCR suggested that RDTs may actually be more sensitive than expert slide reading [36]. However, RDTs are not able to replace microscopy as the gold standard for malaria diagnosis, they can be used as complementary technique in situation where experienced personnel are not available, in order to perform a rapid diagnosis, which can a few hours later be confirmed or corrected by microscopy or PCR.

The areas selected for this study, although distant from main cities of Colombia, benefit from electricity, basic laboratory facilities and are reachable by road. They all had sufficient conditions for proper storage of the test kits. Deki Reader batteries could be recharged easily when needed and communication infrastructure for mobile and land telephone were both available.

TBS microscopy presented serious limitations, with factors such as variability in techniques of blood film preparation, staining, reading standards, and most importantly, highly dependent on the level of expertise of microscopists. In the present study, laboratory technicians had training and experience above the standard level of field microscopist. Despite their university training, the significant loss of information for the final analysis (384 = 17.5%), most of which (238 = 10.8%) presented major violation of RDT manufacturer’s recommendation, indicated that although constant monitoring was available, the two-day training may have been insufficient. The PI and study coordinators had permanent access to the database via internet portal especially designed for the study and were able to monitor the progress of the study and to review images of the RDTs which allowed them to redirect training efforts to the areas where images suggested RDT mishandling. Device users in the field were able to perform the data collection using the touch screen and processing and interpretation of RDTs without major problems. Likely, due to software malfunction early in the course of the study, data from 37 samples (1.7%) were not recorded in the device nor transmitted to the database.

Although some places in remote endemic regions do not yet have reliable mobile phone connectivity, the device saves all data which can be transmitted later under good connections to mobile networks. The data and images displayed in the portal created for this study allowed to follow up closely the performance of field operators. It has also demonstrated the potential to be greatly beneficial to health programme managers by providing a tool to perform remote QA monitoring of field activities. The ability to assist in POC diagnosis, and simultaneously capture and make immediately available pivotal epidemiological data at source of origin makes this a very attractive tool to enhance epidemiological surveillance, strengthen health care systems, and contribute to global mapping efforts of malaria and other infectious diseases [37-39]. The information collected, along with the correspondent RDT image, was sent immediately from remote areas to a database, being part of the Fio Cloud services with 100% fidelity.

Although the function of the Deki Reader is to interpret the presence or absence of a test line signal, independent of the analyses that are finally deposited on it, these results could not be extrapolated to other RDTs. Important differences in RDT clinical diagnostic performance have been well documented, and therefore the results presented here are only applicable to SD Bioline Malaria Antigen Pf/Pv RDT. Nevertheless, the performance of the Reader should be independent to the underlying immunochemistry and thus should provide similar performance characteristics for other RDTs using the same immunochromatographic test.

## Conclusions

The diagnostic performance of the Deki Reader was comparable to visual interpretation of RDTs (concordance rate >98%) for both malaria species. Providing standardized automated interpretation of RDTs at POC in remote areas, in addition to almost real-time reporting of cases and enabling quality control would greatly benefit large-scale implementation of RDT-based malaria diagnostic programmes.

## Competing interests

The authors declare that they have no competing interests. SF and MC are employees of Fio Corporation.

## Authors’ contributions

SH, MA-H and SF conceived and designed the study. SH, MA-H, AV and SF wrote the manuscript. MC and JPQ collected and monitored the data, and provided comments on the manuscript. All authors read and approved the final manuscript.
